# Transfer of the left-side bias effect in perceptual expertise: The case of simplified and traditional Chinese character recognition

**DOI:** 10.1371/journal.pone.0194405

**Published:** 2018-04-02

**Authors:** Tianyin Liu, Su-Ling Yeh, Janet H. Hsiao

**Affiliations:** 1 Department of Psychology, The University of Hong Kong, Hong Kong SAR, China; 2 Department of Psychology, National Taiwan University, Taipei, Taiwan; Nagoya University, JAPAN

## Abstract

The left-side bias (LSB) effect observed in face and expert Chinese character perception is suggested to be an expertise marker for visual object recognition. However, in character perception this effect is limited to characters printed in a familiar font (font-sensitive LSB effect). Here we investigated whether the LSB and font-sensitive LSB effects depend on participants’ familiarity with global structure or local component information of the stimuli through examining their transfer effects across simplified and traditional Chinese scripts: the two Chinese scripts share similar overall structures but differ in the visual complexity of local components in general. We found that LSB in expert Chinese character processing could be transferred to the Chinese script that the readers are unfamiliar with. In contrast, the font-sensitive LSB effect did not transfer, and was limited to characters with the visual complexity the readers were most familiar with. These effects suggest that the LSB effect may be generalized to another visual category with similar overall structures; in contrast, effects of within-category variations such as fonts may depend on familiarity with local component information of the stimuli, and thus may be limited to the exemplars of the category that experts are typically exposed to.

## Introduction

**Fig 1:**
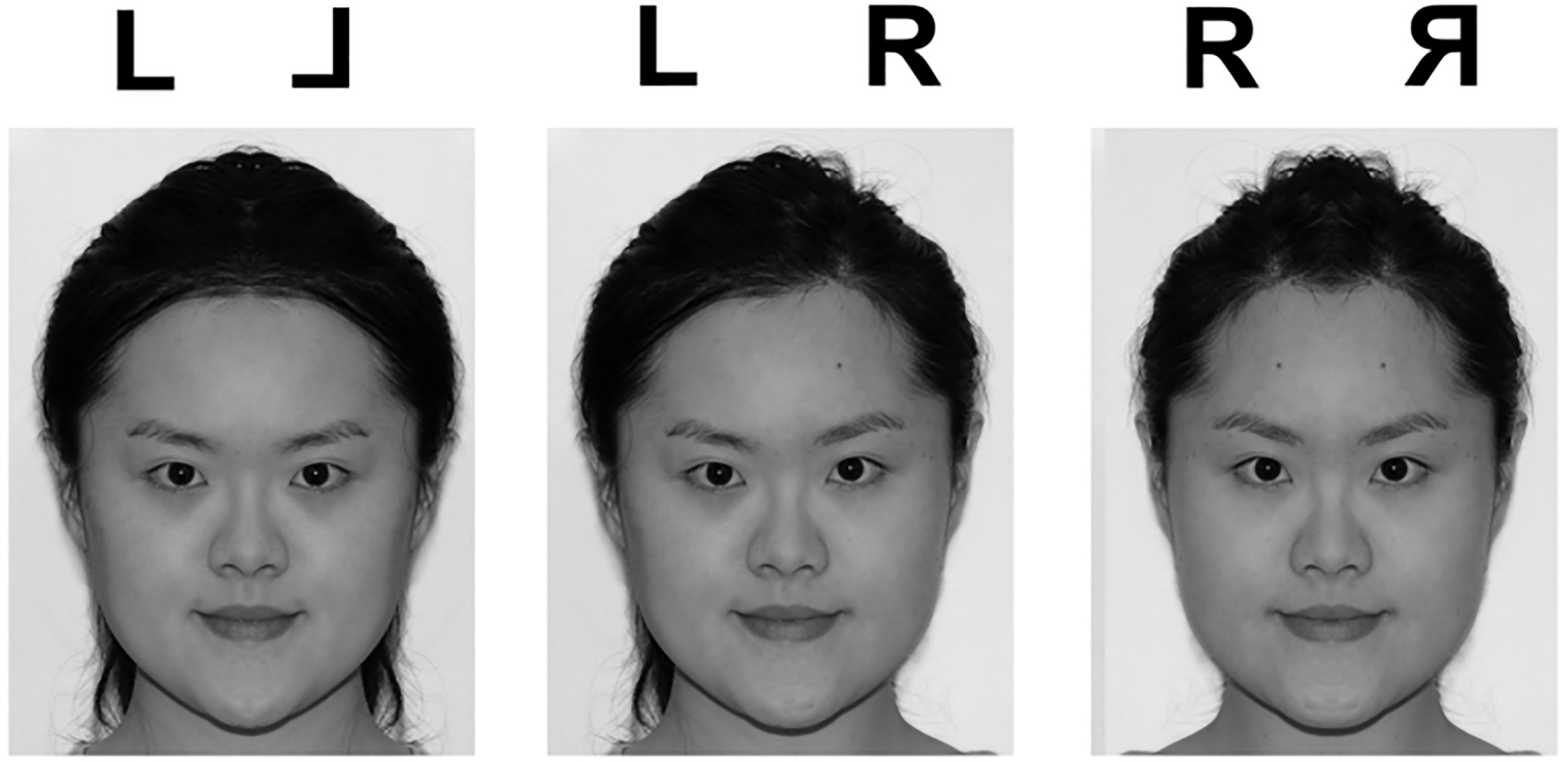
Examples of composite faces created from the original face. The left-left composite face looks more similar to the original face than the right-right composite face.

Mirror symmetry is a salient characteristic of many natural objects, such as patterns on the wings of butterflies, and human faces to a large extent [1]. The human visual system has developed remarkable efficiency in extracting this bilateral symmetry information from only part of the visual input. For instance, when we perceive faces, which are almost mirror symmetrical, our perception appears to be dominated by one half of the faces (from the viewer’s perspective): the left half. A consistent left-side bias (LSB) in the perception of faces has been found: humans have a tendency to judge the left-left composite face to be more representative of the original face than the right-right composite face [2] (see Fig 1 for example, and refer to method section for consent of the participant). This effect has been suggested to be an indicator of the right hemisphere (RH) dominance in face processing, and cannot be accounted for alone by scanning habits developed through reading [3] or one half of the face being more expressive than the other [4]. In addition to identity judgments, similar LSB effects have been found in other face processing tasks, including gender [5] and emotion state judgments [6]. LSB in face perception can be modulated by experience and exposure. For example, 5-year-old children who had no or limited exposure to infant faces showed LSB for adult faces but not for infant faces, and adult participants showed larger LSB for adult faces over infant faces [7]. This result suggested that LSB in visual perception might be a perceptual expertise marker. However, previous studies in this LSB effect have focused on face perception. Although individuals may differ in experiences with faces of different races and ages, it is difficult to recruit novices of face processing for direct comparisons between experts and novices. This problem can be overcome easily if word stimuli are used, as there are many novices of a particular written language.

Chinese is spoken by around one-fifth of the world’s population, and written Chinese is one of the oldest logographic systems still in use. In Chinese orthography, each character has a square-like configuration, and is constructed with three hierarchical levels of organization, namely stroke, component, and global structure of the whole character [8]. Strokes are simple local features such as dots, lines, curves, etc., and they are well defined in Chinese characters. The next perceptual level of Chinese character organization is component/radical, which is formed by a number of strokes, and is also arguably the smallest recognition unit of Chinese characters [9]. A majority of Chinese characters are compound characters made of at least two components, these components are arranged at various positions and thus form the structure of the character, and there are in total five types of forms including left-right, top-bottom, L-shaped, P-shaped, and enclosed [10]. It has been suggested that Chinese character recognition resembles face recognition in some aspects [11]. For instance, both faces and Chinese characters have a homogenous shape, are processed at the individual level, are learnt in an upright orientation, and are recognized regardless of perceptual variations (e.g., expressions/fonts). Chinese orthographic processing is found to be right-lateralized in the visual system of the brain as compared with alphabetic language processing [12], similar to face recognition, although some have suggested bilateral involvement [13]. In particular, similar to faces, some Chinese characters have a mirror-symmetric configuration with some asymmetric features due to shapes of the strokes in some fonts. In contrast to faces, there are many novices of Chinese character recognition available. Thus, these Chinese mirror-symmetric characters provide a unique opportunity for examining expertise effects in LSB in visual perception. Indeed, using these characters as the stimuli, it has been found that expert Chinese readers demonstrate LSB whereas novices do not, suggesting that LSB may be an expertise marker for visual object recognition [14].

**Fig 2:**
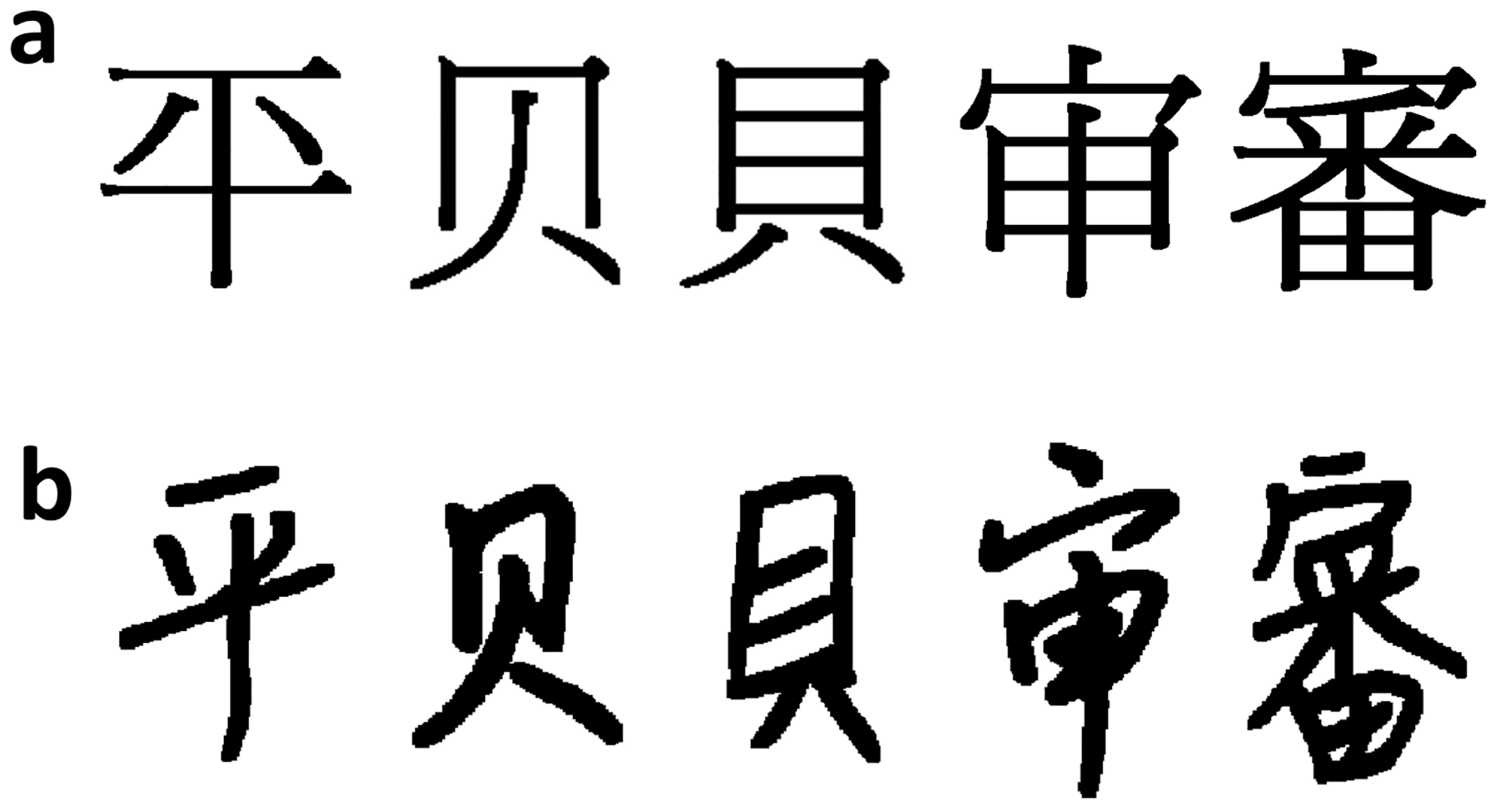
Examples of mirror symmetrical Chinese characters. Same set of characters in (a) Ming Font and (b) Feng Font.

A recent study further revealed that this LSB effect in Chinese character perception depends on readers’ familiarity with the font of the characters, but does not depend on readers’ writing experience [15]. More specifically, it was found that expert Chinese readers who were able to write Chinese characters fluently (i.e., Writers) and those who had limited writing experience (i.e., Limited-writers) did not differ in the LSB effect. Nevertheless, in both groups, the LSB effect was only observed in characters in a familiar font (e.g., Ming, Fig 2a) but not in characters in an unfamiliar font (e.g., Feng; Fig 2b). These findings suggest that the LSB effect, as a perceptual expertise marker in visual object processing, is sensitive to within-category variations such as fonts but insensitive to writing/sensorimotor experience.

In the literature on visual expertise, sensorimotor experience such as writing or drawing has been shown to lead to reduced holistic processing effects in visual recognition. For example, face artists showed less holistic face processing than ordinary observers [16]. A recent computational modeling study suggests that this effect may be due to engagement of local attention in face artists when drawing faces [17]. Similarly, Chinese readers who had extensive writing experience showed reduced holistic processing in perceiving Chinese characters [15], adding evidence to the association between motor experience and local attention. Since the LSB effect in expert Chinese character processing is insensitive to readers’ writing experience, this phenomenon suggests that the LSB effect may not rely on readers’ familiarity with local featural information of the stimuli. In contrast, it may depend more on readers’ familiarity with global structural information of the stimuli.

The font-sensitive LSB effect among expert readers is consistent with previous research findings of font tuning effects in reading expertise [18]: in printed word recognition, font information, although irrelevant to the task, is processed by readers automatically. In this study, Gauthier et al. asked both Chinese-English bilinguals and English monolinguals to learn the association between keys on a keyboard and Chinese characters/Roman letters, and then perform an identification task of Chinese characters/Roman letters in either same or mixed fonts. It was found that changes in font regularity of Roman letters affected both groups, while identification of Chinese characters in mixed fonts slowed down Chinese-English bilinguals but not English monolinguals as compared with their baseline performance. This effect showed that expert readers were sensitive to font variations in the mixed font condition whereas novice were not, suggesting that experts were more sensitive to within-category exemplar variations than novices. More specifically, since exemplars of a character in different fonts typically share a similar overall structure but differ in features of local strokes, expert readers’ higher sensitivity to font variations as compared with novices may be related to their better ability in processing local information of the stimuli. Thus, the font-sensitive LSB effect may rely more on readers’ familiarity with local featural information of the stimuli.

**Fig 3:**
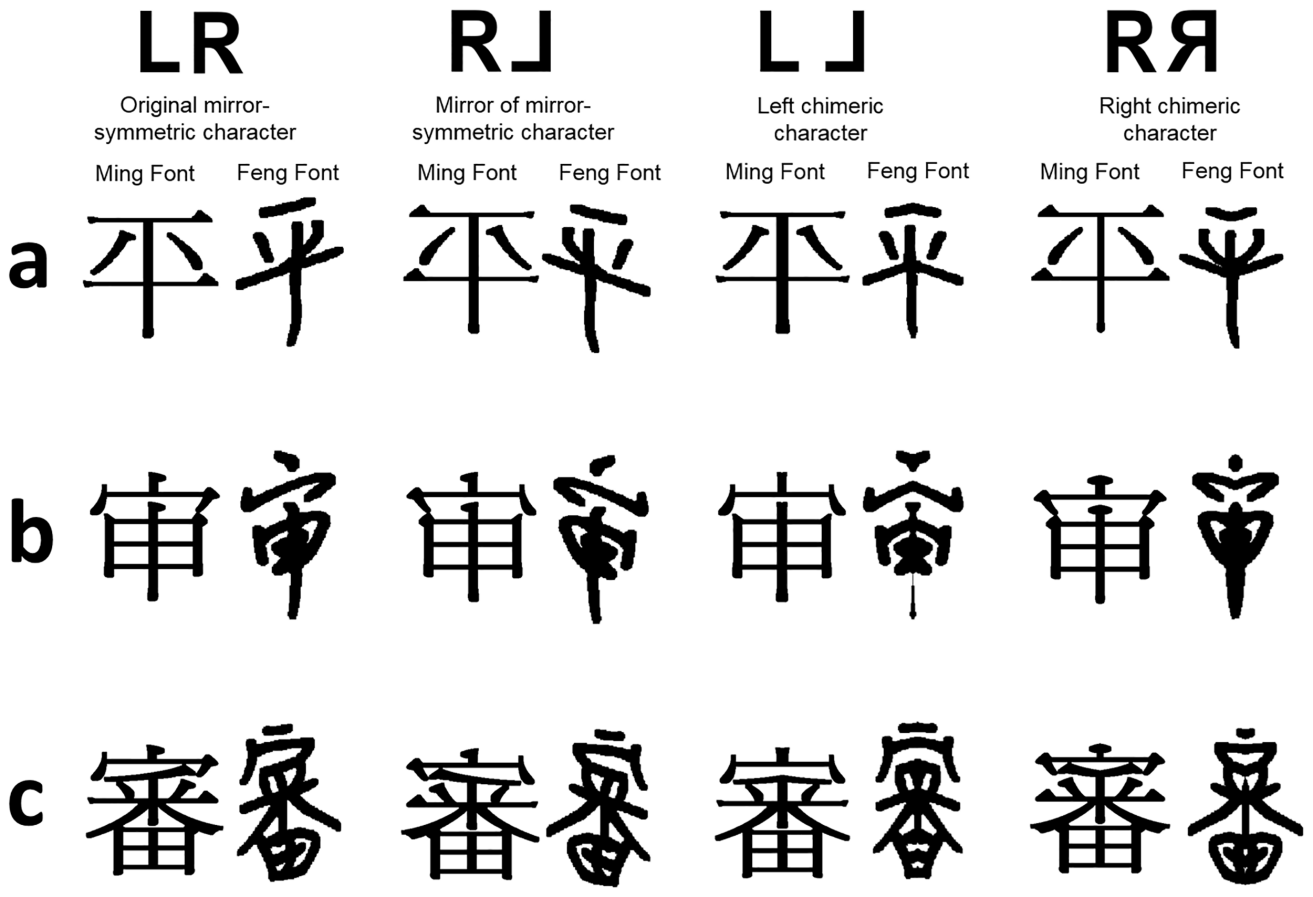
Examples of left (LL) and right (RR) chimeric and mirror (RL) Chinese characters created from original mirror-symmetric characters (LR). Characters are presented in both Ming and Feng fonts in all three character types: (a) shared, (b) simplified, and (c) traditional characters.

Accordingly, here we aim to investigate whether the LSB effect and the font-sensitive effect in LSB depend on participants’ familiarity with global structural or local featural information of the stimuli through examining their transfer effects across simplified and traditional Chinese scripts. The two Chinese scripts share similar overall structures but differ in the complexity of local components in general, providing a unique opportunity for this examination. More specifically, there are currently two Chinese writing systems in use in Chinese speaking regions, namely simplified and traditional Chinese. Regions including Mainland China, Singapore, and Malaysia use the simplified script, while Hong Kong and Taiwan continue to use the traditional script. Moreover, the simplification process did not apply to all characters; among the most frequently used 3,500 characters, around 40% were simplified and have approximately 22.5% fewer strokes than the traditional counterparts [19]. The remaining 60% of the characters stayed the same; i.e., they are shared between the simplified and traditional Chinese writing systems. The basic emphases in the simplification process of traditional Chinese characters were: to simplify common radicals of the characters (e.g., radical “言” was simplified as “讠”, and applied to characters containing this radical, “討” to “讨”, “話” to “话”, etc.), to remove elements within the characters (e.g., “愛” to “爱”, and “審” to “审”), and to combine some homophones and eliminate redundant characters (e.g., “範” and “范” were combined into the latter, and the former was eliminated) [20]. Thus, the general structures of traditional Chinese characters remained largely the same as their simplified counterparts (e.g., with a left-right or top-bottom configuration, and general shape of the components), but with different component features and complexity (i.e., more strokes; see Fig 3 for examples. Fig 3b shows the simplified version of the character in Fig 3c with a similar structure but less detail). Due to the similarity in overall structures between simplified and traditional characters, simplified and traditional Chinese readers are generally accurate in recognizing characters from both scripts; however, they may have difficulty in writing the traditional and simplified Chinese characters from memory respectively because of the differences in local features [21].

The existence of simplified and traditional Chinese scripts creates a unique opportunity for us to examine whether LSB and the font-sensitive effect in LSB depend on participants’ familiarity with global or local information of the stimuli. More specifically, here we aim to investigate whether there is a transfer of LSB and, if so, whether the transfer of LSB and the font-sensitive LSB effect relies on the similarity in overall structure or in local features. We hypothesize that the transfer of LSB may depend more on the similarity in overall structure between two stimulus categories. In contrast, since previous research showed increased font sensitivity in reading expertise [18], the font-sensitive LSB effect may rely more on the familiarity with distinctive local features. This speculation is consistent with the global precedence hypothesis [22], which argues that the global form of a visual stimulus is unavoidably recognized before the local forms; thus, in visual expertise acquisition, familiarity with the global form may precede familiarity with the local information. Therefore, due to the similar overall structures between simplified and traditional Chinese scripts, simplified and traditional Chinese readers may transfer LSB to the processing of the scripts they are less familiar with, but may only show the font-sensitive LSB effect in the script they are familiar with due to the lack of sensitivity to font variations in reading the script they are not familiar with.

## Methods

Here we adopted Hsiao and Cottrell’s procedure [14] to examine the LSB and font-sensitive LSB effect in traditional Chinese readers from Taiwan, simplified Chinese readers from Mainland China, and non-Chinese readers (novices) as controls. This study was approved by Human Research Ethics Committee for Non-Clinical Faculties of the University of Hong Kong (Reference No. EA220114), and informed consent forms were signed by all participants before taking part in this study.

### Participants

One female experimenter was invited to take a portrait of herself to create Fig 1. This individual in this manuscript has given written informed consent (as outlined in PLOS consent form) to publish these case details. Thirty native traditional Chinese readers (14 males, 16 females) in Taiwan, 30 native simplified Chinese readers (8 males, 22 females) from Mainland China, and 30 non-Chinese readers (novices, 15 males, 15 females) whose first languages were alphabetic languages participated in the study. The traditional Chinese participants were all students at National Taiwan University, while the simplified Chinese readers and non-Chinese readers were all students at University of Hong Kong. Note that the language of instruction at the University of Hong Kong is English. All simplified Chinese readers in the study had stayed in Hong Kong for less than half a year by the time they were recruited (average length of stay = 5.3 months), and same for the novices of Chinese (average length of stay = 4.6 months). The simplified Chinese readers had an average age of 22.20 (*SE* = .63), traditional Chinese readers 23.60 (*SE* = .52), and novices 22.03 (*SE* = .37). Note that they were marginally different in age when recruited (*F* (2, 87) = 2.73, *p* = .07) as a result of random sampling: the traditional Chinese readers were about one-year older than the other two groups. Similarly, the simplified Chinese readers had on average 15.64 (*SE* = .45) years of education, traditional Chinese readers 16.70 (*SE* = .40), and novice 15.37 (*SE* = .37), and they were marginally different in years of education when recruited (*F* (2, 87) = 2.98, *p* = .06). The three groups all had normal or corrected-to-normal vision and were right-handed according to Edinburgh Handedness Inventory [23].

### Materials

One-hundred-and-twenty mirror-symmetric Chinese characters (see S1 Appendix) with medium to high character frequency (from 105 per million to 1068 per million) [24] were used. Among them, 40 were traditional characters (mean number of strokes = 13.95, *SE* = .67; character frequency between 105 and 941, median = 254), 40 were the simplified counterparts of the traditional characters (mean number of strokes = 8.13, *SE* = .50; character frequency between 105 and 1068 per million, median = 321), and 40 were shared between two Chinese scripts (mean number of strokes = 7.68, *SE* = .34; character frequency between 107 and 1043 per million, median = 272). The three types of characters were matched in character frequency (*F*(2, 117) = .49, *p* = .51). Simplified and shared characters were matched in visual complexity defined by number of strokes (*F*(1, 78) = .56, *p* = .46), whereas traditional characters were more complex than both shared (*F*(1, 78) = 68.81, *p<* .01) and simplified characters (*F*(1, 78) = 48.37, *p<* .01). We created chimeric characters based on these original mirror symmetrical characters in the same fashion as chimeric faces were created. More specifically, to create a left chimeric character (LL), an original mirror-symmetrical character was firstly vertically split from the middle, and then a left chimeric character was created by concatenating the original left half with the mirror image of that left half. The right chimeric character (RR) was constructed in a similar way (see Fig 3 for examples).

Each original mirror-symmetric character (120 in total: 40 shared, 40 simplified, and 40 traditional Chinese characters) was presented twice in Ming and Feng fonts respectively. In each trial, an original character was shown together with the LL and RR forms of it, and thus in total there were 240 trials (120 characters x 2 fonts) in the experiment. In half of the trials the mirror images of the stimuli were used to counterbalance possible featural differences between the two sides of the characters that may be confounded with any perceptual bias effect. The mirror images were created by flipping the original stimuli along the vertical midline (see Fig 3 for examples). This is a common technique used in previous studies examining left-side bias in face perception to counterbalance possible featural differences between the two sides of the face stimuli that may be confounded with the left-side bias effect. Since the stimuli used in this study were mirror-symmetric characters, the mirror images of the characters had the same character identity as the original characters and only differed in stroke features. Note that no non-character was used in the current study. For each stimulus, the presentation of the original/mirror-image forms was counterbalanced across participants and fonts, e.g., if a character was presented in Feng font in its original form, the same character would be presented in its mirror-image in Ming font.

To examine whether the traditional and simplified Chinese character pairs used here were indeed generally perceived as having similar overall structures but differ in local features, we carried out a separate survey with 20 Chinese novices whose first languages were alphabetic languages as participants (10 male, 10 female; 15 native English, 2 German, 1 French, 1 Portuguese, and 1 Czech speakers). All participants had none or very limited exposure to Chinese. They were asked to rate the similarities of the 40 pairs of simplified and traditional Chinese characters used in the LSB task in terms of global structure and local features. The simplified and traditional Chinese characters in a pair was present simultaneously, and participants rated their similarity in Linkert scale from 1 to 9, with 1 being completely different, and 9 being exactly the same. The character pairs were presented in both Ming and Feng fonts, and thus there were 80 trials in total. The results confirmed that the character pairs were perceived to be more similar in global structure over local features in both Ming (*t*(39) = 10.79, *p* < .001) and Feng font (*t*(39) = 12.04, *p* < .001). The average similarity score in global structure was 5.66 (*SE* = .62) in Ming font, and 5.29 (*SE* = .62) in Feng font; while the similarity score in local features was 4.50 (*SE* = .64) in Ming font, and 4.11 (*SE* = .55) in Feng font.

### Design

The design had two within-subject variables: character type (shared vs. simplified vs. traditional), and font type (Ming vs. Feng), resulting in six sub-categories of stimuli corresponding to different characters type and font combinations; and a between-subject variable: group (Mainland simplified Chinese readers vs. Taiwan traditional Chinese readers vs. novices of Chinese). The dependent variable LSB in each sub-category was the preference for the left chimeric character, calculated as the number of trials that the left chimeric character was judged more similar to the original one divided by total number of trials in each sub-category (i.e., 40 trials).

### Procedure

#### Left-side bias

**Fig 4:**
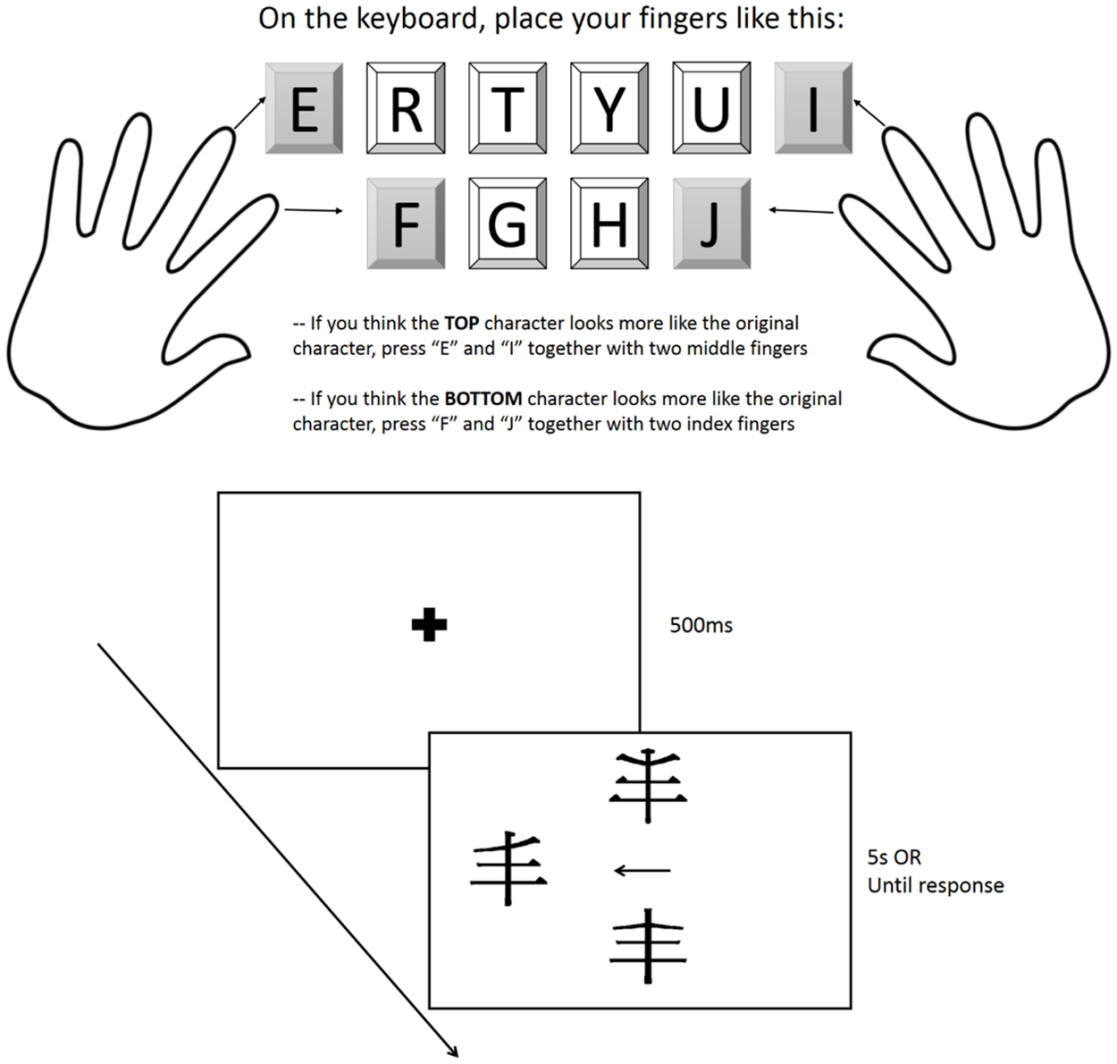
Placement of fingers and procedure in a left-side bias task.

In each trial, the original, left- and right-chimeric characters were of the same size (1.5 degrees of visual angle with 50 cm viewing distance) and were shown simultaneously on the screen. The original character was either to the left or the right side of the screen, with an arrow in the middle to indicate its position. The two chimeric characters were presented above and below the arrow about 2.5 degrees of visual angle away respectively, and the position of the two chimeric character choices was counterbalanced across stimuli and across participants. Each trial started with a 500ms central fixation, followed by the character presentation. Participants were asked to follow the arrow to look at the original character, and then judge which chimeric character looked more similar to the original by pressing keys with both hands. More specifically, following the finger placement of a standard QWERTY keyboard layout, they pressed “E” (left) and “I” (right) together with two middle fingers for the top character, and pressed “F” (left) and “J” (right) with two index fingers together for the bottom character (Fig 4). This design was to control for any lateralization effect that may be induced by responding with one hand. The stimuli, one original and two chimeric characters, were presented for at most five seconds or until the participants’ response (Fig 4). Two hundred and forty trials were organized in three blocks, each containing only one type of characters (i.e., shared, simplified or traditional characters, in both Ming and Feng fonts). Within each block, the presentation order of the 80 stimuli was randomized. The presentation order of the three blocks was counterbalanced across participants.

#### Reading and writing performance

To examine Taiwan traditional Chinese readers’ and Mainland simplified Chinese readers’ familiarity with the simplified and traditional scripts, tasks adopted from Tso et al. [15] were administered. A character naming task was used to assess participants’ reading ability, and a word dictation task was used to examine their writing ability. All reading and writing assessment tasks were carried out after the completion of the left-side bias task. Breaks were given between tasks, and on average it took participants 30 minutes to finish the reading and writing tasks.

In the character naming task, 120 Chinese characters were used, among them 40 were simplified characters, 40 were the corresponding traditional version of the simplified characters, and the remaining 40 were shared characters. These characters were not used in the left-side bias task. All characters selected for the naming task were of medium to high frequency (ranging from 28 per million to 1316 per million) [25] and were matched in relative frequency across the script types (*F*(2, 117) = 1.69, *p* = .19). In terms of visual complexity, the traditional characters had significantly more strokes than their simplified counterparts (*t*(39) = 10.92, *p* < .01). In each experimental trial, a central fixation was presented first for 500 ms, followed by a character occupying approximately 1.5 degrees of visual angle at the center of the screen. Participants were asked to read the character out in front of a microphone. The onset of their pronunciation was detected by a microphone attached to a serial response box. Their response time was recorded as the duration between the onset of the character presentation and the onset of the pronunciation. The experimenter then pressed buttons on a response box to record the accuracy of participants’ response and initiate the next trial.

In the word dictation task, 40 Chinese characters were used, among them 20 were shared Chinese characters and 20 traditional/simplified, all selected from the character naming task. Because a single Chinese character has around 11 homophones on average [26], to avoid ambiguity, each character was concatenated with a second character to compose a two-character word for dictation. All words were of medium to high frequency (from 162 per million to 1037 per million) [25] and were matched in relative word frequency across the three character types (*F*(2, 57) = 2.77, *p* = .11). Participants listened to the words presented in a female voice. The audio recordings of the words were presented by a computer in a random order. Participants wrote down each word in their own script first and then in the other script, even if they thought the characters were the same in the two scripts. If they did not know how to write a character, they indicated it by putting a cross on the space. In each trial, after the words “get ready” presented on the screen for 500ms, participants were presented with a stimulus. They then pressed a button on a serial response box to indicate whether they knew how to write it or not. After writing the word in both scripts, they pressed a button to indicate completion and to start the next trial. Their accuracy of writing the first character of each word was assessed.

## Results

The results of the reading and writing tasks revealed that simplified and traditional Chinese readers in general could read but not write the script they were not familiar with fluently. In the reading tasks, there was no significant difference in their naming accuracy in shared characters (*F*(1, 58) = .66, *p* = .42). Although simplified Chinese readers had higher accuracy than traditional Chinese readers in naming simplified characters (*F*(1, 58) = 55.20, *p* < .001, *η*_*p*_^*2*^ = .49), and vice versa in naming traditional Chinese characters (*F*(1, 58) = 10.09, *p* < .01, *η*_*p*_^*2*^ = .13), they all achieved high proficiency as revealed by the over 95% average accuracy (Table 1). In contrast, in the dictation task, simplified Chinese readers performed better than Taiwan traditional Chinese readers in writing simplified characters (*F*(1, 58) = 237.74, *p* < .001, *η*_*p*_^*2*^ = .80), and vice versa in writing traditional characters (*F*(1, 58) = 732.46, *p* < .001, *η*_*p*_^*2*^ = .93; see Table 1). They had similar performance in writing shared Chinese characters (*F*(1, 58) = 1.84, *p* = .18). For a summary of their performance in reading and writing tasks, please refer to Table 1.

**Table 1:** Mainland simplified Chinese readers and Taiwan traditional Chinese readers’ performance in reading and writing tasks.

Task	Script Type	Mainland Simplified Chinese readers		Taiwan Traditional Chinese readers		Group comparison	
		Accuracy mean (SD)	RT (sec) mean (SD)	Accuracy mean (SD)	RT (sec) mean (SD)	Accuracy	RT
**Naming**	Shared	.99 (.00)	.28 (.02)	.99 (.00)	.38 (.02)	*p* = .42	*p* < .01
	Simplified	1.00 (.00)	.28 (.02)	.95 (.01)	.42 (.02)	*p* < .001	*p* < .01
	Traditional	.98 (.01)	.30 (.01)	.99 (.00)	.38 (.02)	*p* < .01	*p* < .01
**Dictation**	Shared	.98 (.01)		1.00 (.00)		*p* = .18	
	Simplified	.99 (.01)		.37 (.04)		*p* < .001	
	Traditional	.18 (.04)		1.00 (.00)		*p* < .001	

As for their performance in the chimeric character judgment task, an analysis of variance (ANOVA) with mixed design was carried out, with LSB as the dependent variable, character type and font as two within-subject variables, and reader group as a between-subject variable. ANOVA on subject analysis revealed a significant main effect of font (*Fs*(1, 87) = 5.66, *p* < .05, *η*_*p*_^*2*^ = .06), a significant two-way interaction between font and group (*Fs*(2, 87) = 4.25, *p* < .05, *η*_*p*_^*2*^ = .09), and a significant three-way interaction between character type, group, and font (*Fs*(4, 174) = 3.27, *p* < .05, *η*_*p*_^*2*^ = .07). A by-item analysis was also performed using ANOVA, with LSB as the dependent variable, font and reader group as two within-subject variables, and character type as a between-subject variable. It revealed significant main effects of font (*Fi*(1, 117) = 5.93, *p* < .05, *η*_*p*_^*2*^ = .05) and reader group (*Fi*(2, 234) = 5.06, *p* < .05, *η*_*p*_^*2*^ = .04), a significant two-way interaction between font and group (*Fi*(2, 234) = 4.69, *p* < .05, *η*_*p*_^*2*^ = .04), and a significant three-way interaction between character type, group, and font (*Fi*(4, 234) = 5.03, *p* < .01, *η*_*p*_^*2*^ = .08). The results were similar to those of the by-subject analysis.

To better understand this three-way interaction, we further examined participants’ performance in the three character-type conditions separately.

### Shared Chinese characters

**Fig 5:**
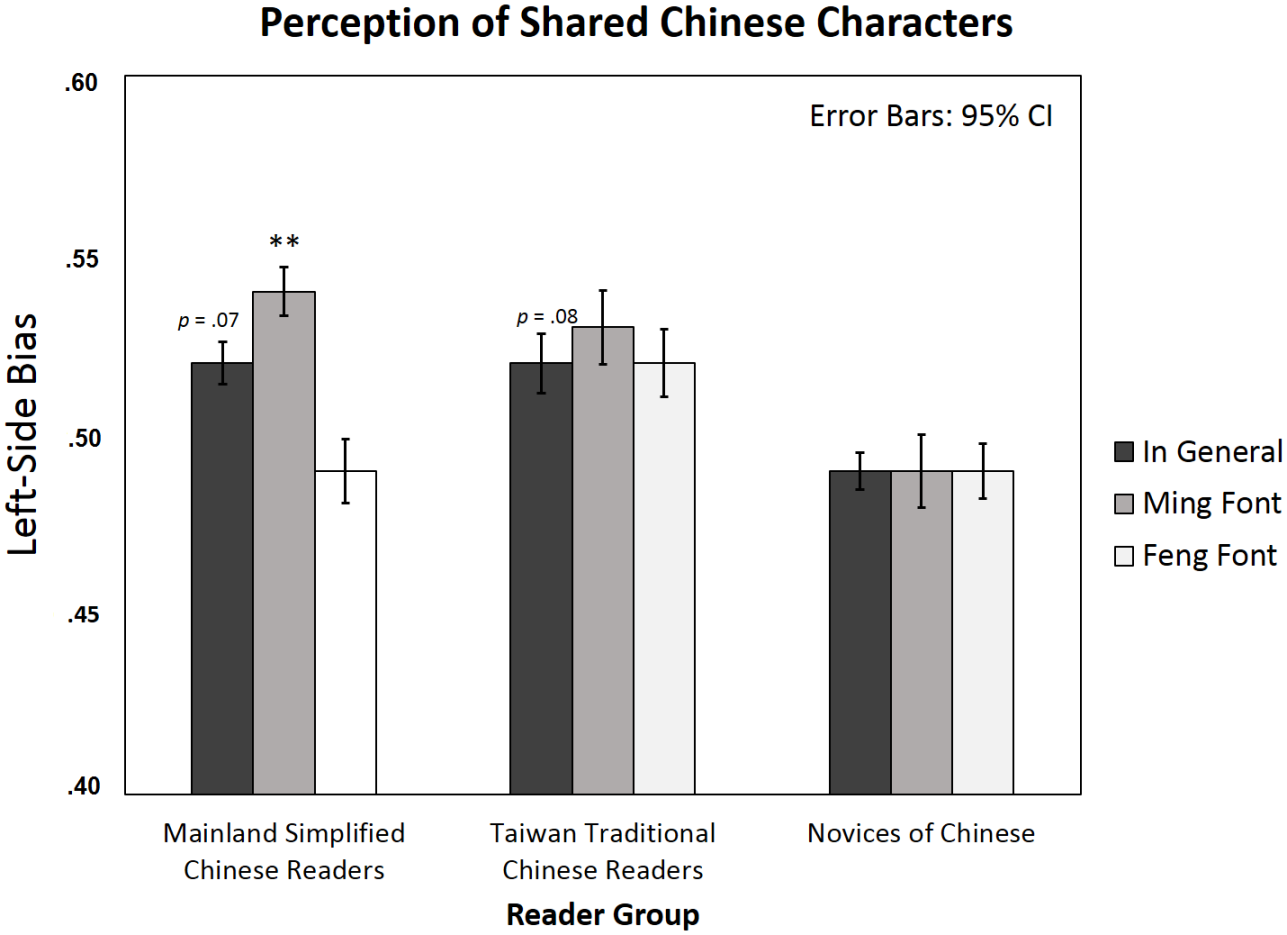
Left-side bias in perceiving shared characters in simplified and traditional Chinese readers and novices of Chinese (means and 95% CIs). Asterisks indicate significant Left-side bias (one sample t-test against the chance level .5) in each condition separately; * *p* < .05; ** *p* < .01.

The results showed a significant interaction between font and group (*Fs*(2, 87) = 3.20, *p* < .05, *η*_*p*_^*2*^ = .07; *Fi*(2, 78) = 4.75, *p* < .05, *η*_*p*_^*2*^ = .11) and a marginal main effect of group (*Fs*(2, 87) = 2.69, *p* = .07; *Fi*(2, 78) = 3.22, *p* = .05). When we examined the difference in LSB among the groups, both Chinese reader groups showed a marginal LSB effect, as compared with the chance level .5, in the perception of shared characters in general (Mainland simplified Chinese readers, mean LSB = .52, *t*(29) = 1.88, *p* = .07, *d* = .34; Taiwan traditional Chinese readers, mean LSB = .52, *t*(29) = 1.82, *p* = .08, *d* = .33. The two Chinese reader groups did not differ in the LSB effect, *F*(1, 58) = .30, *p* = .59). In contrast, novices of Chinese did not show LSB (*t*(29) = -1.43, *p* = .17). When we examined the font-sensitive LSB effect in the three reader groups separately, simplified Chinese readers demonstrated a strong font-sensitive LSB effect (*t*(29) = 3.41, *p* < .01, *d* = .62): there was a strong LSB in Ming font (mean LSB = .54, *t*(29) = 3.57, *p* < .01, *d* = .65) but not in Feng font (mean LSB = .49, *t*(29) = -.95, *p* = .35). In contrast, there was no font-sensitive LSB effect observed in either traditional Chinese readers (*t*(29) = .17, *p* = .87) or novices (*t*(29) = -.19, *p* = .85; see Table 2 and Fig 5).

**Table 2:** Left-side bias in perceiving shared, simplified, and traditional Chinese characters in simplified and traditional Chinese readers and novices of Chinese.

Character type	Left-side Bias	Simplified Chinese readers (n = 30)		Traditional Chinese readers (n = 30)		Novices of Chinese (n = 30)	
**Shared Chinese characters**	In general	.52 (.05)	*p* = .07	.52 (.07)	*p* = .08	.49 (.04)	*p* = .17
	Ming font	.54 (.06)	*p* < .01	.53 (.09)	*p* = .14	.49 (.08)	*p* = .45
	Feng font	.49 (.07)	*p* = .34	.52 (.08)	*p* = .12	.49 (.06)	*p* = .57
**Simplified Chinese characters**	In general	.52 (.05)	*p* = .07	.51 (.04)	*p* = .06	.51 (.06)	*p* = .56
	Ming font	.55 (.07)	*p* < .01	.51 (.06)	*p* = .13	.50 (.08)	*p* = .96
	Feng font	.48 (.07)	*p* = .23	.51 (.06)	*p* = .21	.51 (.08)	*p* = .53
**Traditional Chinese characters**	In general	.52 (.06)	*p* < .05	.52 (.04)	*p* < .05	.50 (.08)	*p* = .76
	Ming font	.51 (.08)	*p* = .41	.54 (.07)	*p* < .01	.49 (.11)	*p* = .69
	Feng font	.53 (.08)	*p* < .05	.50 (.06)	*p* = .95	.50 (.09)	*p* = .98

### Simplified Chinese characters

**Fig 6:**
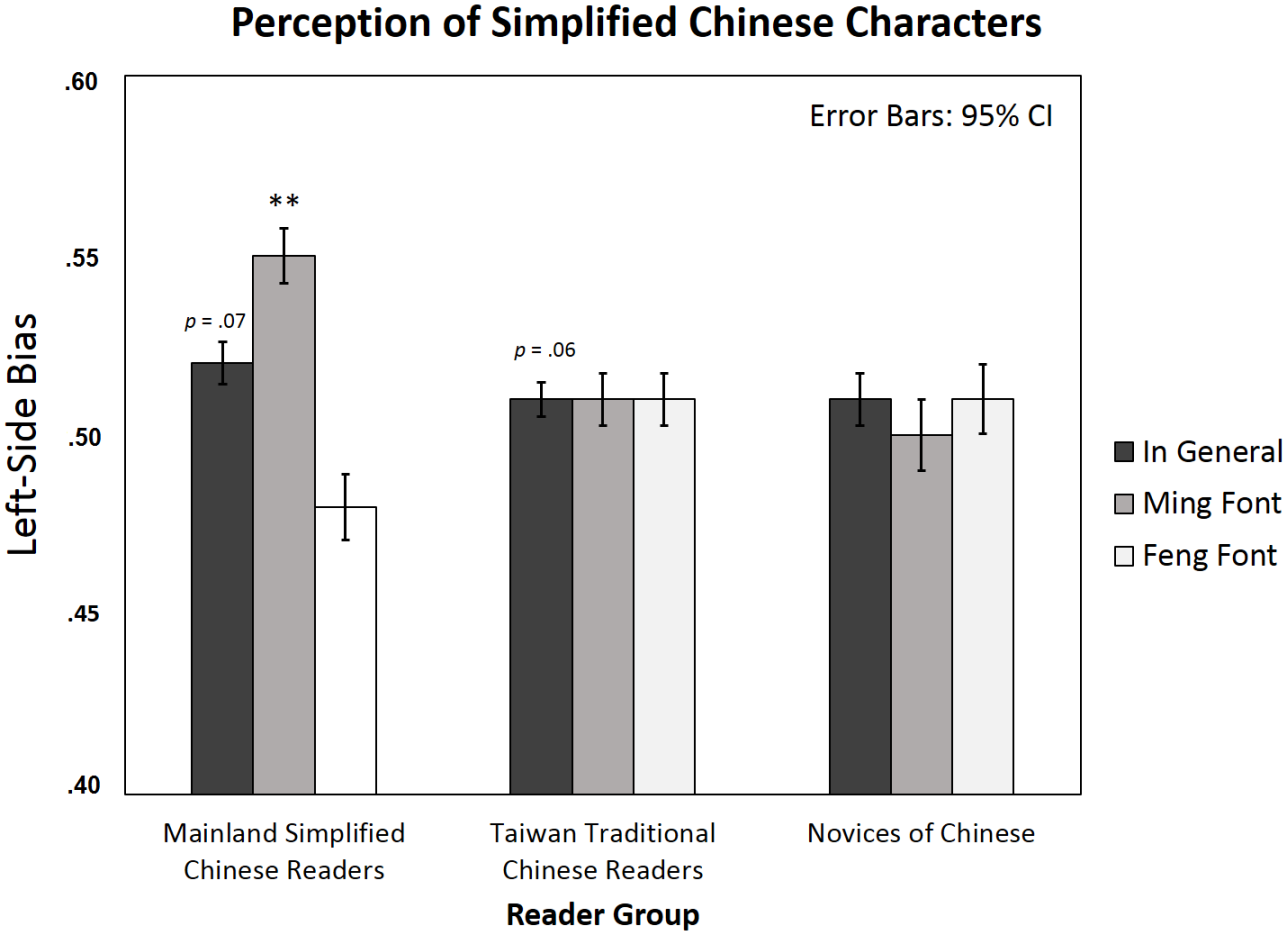
Left-side bias in perceiving simplified characters in simplified and traditional Chinese readers and novices of Chinese (means and 95% CIs). Asterisks indicate significant Left-side bias (one sample t-test against the chance level .5) in each condition separately; * *p* < .05; ** *p* < .01.

There was a significant interaction between font and group (*Fs*(2, 87) = 5.20, *p* < .01, *η*_*p*_^*2*^ = .11; *Fi*(2, 78) = 6.93, *p* < .01, *η*_*p*_^*2*^ = .15), and a marginal main effect of font (*Fs*(1, 87) = 3.37, *p* = .07, *η*_*p*_^*2*^ = .04; significant in by item analysis, *Fi*(1, 39) = 4.56, *p* < .05, *η*_*p*_^*2*^ = .11). Although the main effect of group was not significant (*Fs*(2, 87) = .03, *p* = .86; *Fi*(2, 78) = .65, *p* = .53), both Chinese reader groups had a marginal LSB effect in perceiving simplified Chinese characters (Mainland simplified Chinese readers, mean LSB = .52, *t*(29) = 1.90, *p* = .07, *d* = .35; Taiwan traditional Chinese readers, mean LSB = .51, *t*(29) = 1.93, *p* = .06, *d* = .35. The two Chinese reader groups did not differ in the LSB effect, *Fs*(1, 58) = .03, *p* = .86; *Fi*(1, 39) = .25, *p* = .62), while novices showed no LSB (*t*(29) = .59, p = .56). When we examined the font-sensitive LSB effect in the three participant groups separately, simplified Chinese readers had a significant font-sensitive LSB effect in perceiving simplified characters (*t*(29) = 3.80, *p* < .05, *d* = .69), with strong LSB in Ming font (mean LSB = .55, *t*(29) = 3.86, *p* < .01, *d* = .70) but not in Feng font (mean LSB = .48, *t*(29) = -1.23, *p* = .23). In contrast, neither traditional Chinese readers (*t*(29) = -.02, *p* = .98; Ming font mean LSB = .51, *t*(29) = 1.56, *p* = .13; Feng font mean LSB = .51, *t*(29) = 1.29, *p* = .21) nor novices showed a font-sensitive LSB effect (*t*(29) = -.43, *p* = .67; Ming font mean LSB = .50, *t*(29) = .05, *p* = .96; Feng font mean LSB = .51, *t*(29) = .63, *p* = .53; see Table 2 and Fig 6).

### Traditional Chinese characters

**Fig 7:**
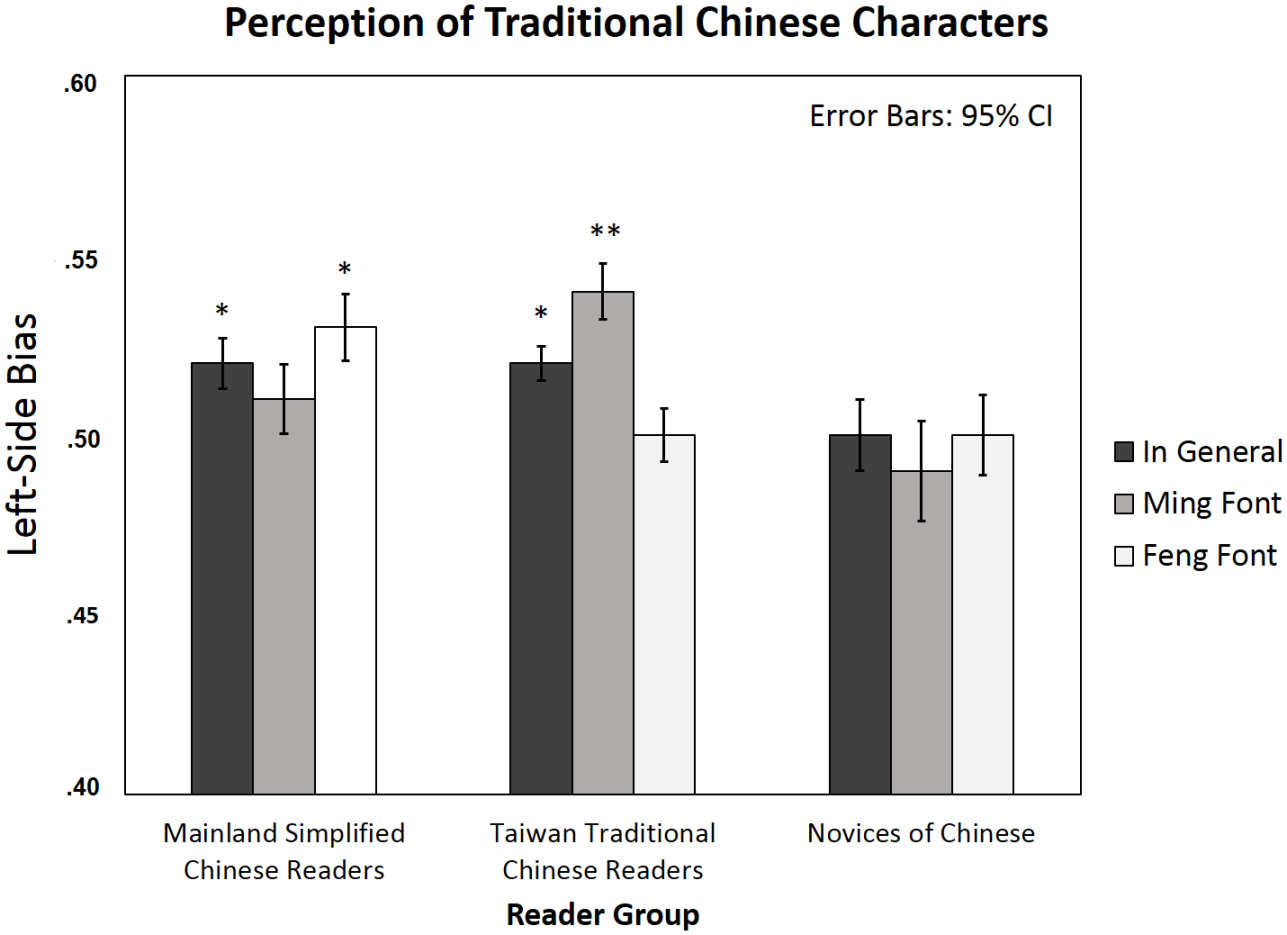
Left-side bias in perceiving traditional characters in simplified and traditional Chinese readers and novices of Chinese (means and 95% CIs). Asterisks indicate significant Left-side bias (one sample t-test against the chance level .5) in each condition separately; * *p* < .05; ** *p* < .01.

No main effect of font (*Fs*(1, 87) = .01, *p* = .75; *Fi*(1, 39) = .43, *p* = .52) or group (*Fs*(2, 87) = 1.54, *p* = .22; *Fi*(2, 78) = 1.92, *p* = .15) was found. Nevertheless, both Mainland simplified Chinese readers (mean LSB = .52, *t*(29) = 2.12, *p* < .05, *d* = .39) and Taiwan traditional Chinese readers (mean LSB = .52, *t*(29) = 2.45, *p* < .05, *d* = .45) showed a significant LSB in perceiving traditional Chinese characters in general (the two groups did not differ significantly in the LSB effect, *F*(1, 58) = .07, *p* = .79), whereas novices did not show LSB (*t*(29) = -.31, *p* = .76). There was a marginal interaction between font and group (*Fs*(2, 87) = 2.54, *p* = .09, *η*_*p*_^*2*^ = .06; significant in by item analysis, *Fi*(2, 78) = 3.37, *p* < .05, *η*_*p*_^*2*^ = .08). Note that when we directly compared the two Chinese reader groups, the interaction between font and group was significant (*Fs*(1, 58) = 5.32, *p <* .05, *η*_*p*_^*2*^ = .09; *Fi*(1, 39) = 6.57, *p <* .05, *η*_*p*_^*2*^ = .14). When we examined the font-sensitive LSB effect in the three participant groups separately by paired sample t-test between Ming font and Feng font LSB, there was a significant font-sensitive LSB effect among traditional Chinese readers (*t*(29) = 2.46, *p <* .05, *d* = .45; Ming font mean LSB = .54, *t*(29) = 3.21, *p* < .01, *d* = .59; Feng font mean LSB = .50, *t*(29) = .07, *p* = .95), but not in simplified Chinese readers (*t*(29) = -1.0, *p* = .33; Ming font mean LSB = .51, *t*(29) = .83, *p* = .41; Feng font mean LSB = .53, *t*(29) = 2.12, *p* < .05, *d* = .39) or in novices (*t*(29) = -.37, *p* = .71; Ming font mean LSB = .49, *t*(29) = -.40, *p* = .69; Feng font mean LSB = .50, *t*(29) = .02, *p* = .98; see Table 2 and Fig 7). Thus, both Chinese reader groups showed a significant LSB effect in general, but only traditional Chinese readers showed the font-sensitive LSB effect.

In summary, both Chinese reader groups demonstrated LSB in the perception of Chinese characters while novices did not, consistent with the previous finding that LSB is a perceptual expertise marker in visual object processing (14, 15). In the perception of shared characters, both simplified and traditional Chinese readers had a similar level of LSB; however, simplified Chinese readers showed a font-sensitive LSB effect while traditional Chinese readers did not. As for simplified and traditional characters, both reader groups had a similar level of LSB; however, they showed a font-sensitive LSB effect (i.e., LSB was observed only for characters in a familiar font but not for those in an unfamiliar font) only in perceiving the character type they were more familiar with. More specifically, in the perception of traditional characters, simplified Chinese readers did not differ from traditional Chinese readers in LSB, but they did not show a similar font-sensitive LSB effect as traditional Chinese readers; vice versa for traditional Chinese readers in the perception of simplified Chinese characters. In all, these results suggest that both groups have transfer effects in the LSB effect in the perception of the script they are not familiar with in general, but have no transfer in the font-sensitive LSB effect.

## Discussion

Previous research revealed that LSB is a perceptual expertise marker of visual object recognition [14]. Our results added to the evidence by showing that both Chinese reader groups demonstrated LSB in the perception of Chinese characters while novices did not. LSB is also sensitive to within-category variations such as font information since it was only observed in characters in a familiar font but not in an unfamiliar font [15]. The existence of simplified and traditional Chinese scripts provides us a unique opportunity to examine transfer effects in LSB and the font-sensitive LSB effect. Simplified and traditional Chinese characters typically have similar overall structures but differ in the visual complexity of local components. Due to the similarities in overall structures, readers might be able to transfer the LSB to the characters in the less familiar script. As for the font-sensitive LSB effect, previous research has suggested that expert readers have a higher sensitivity to font variations than novice readers [18], and thus the font-sensitive LSB effect may not be transferred to a less familiar script.

Consistent with our speculation, we found that simplified Chinese readers were able to transfer the LSB to the perception of traditional characters, and vice versa for traditional Chinese readers, suggesting that LSB in expert Chinese character processing can be transferred across different scripts, possibly due to the similarities in overall structures between the two scripts. However, the two reader groups had different font-sensitive LSB effect: both groups demonstrated a font-sensitive LSB effect in the perception of the character type they were more familiar with, but not in the perception of the character type they were unfamiliar with. This result suggests that the font-sensitive LSB effect is an expertise marker for Chinese character recognition that only emerges in fonts the readers are familiar with. It also mirrors the findings in the face perception literature, where LSB is a small but robust effect in people’s perception of unfamiliar faces (62%) [27, 28], and is much more prominent in judging familiar faces (81%, significantly higher than 62%) [4]. It seems that repetitive exposure to characters in a particular font increases familiarity with the particular visual forms of the characters, and in turn leads to stronger LSB. In contrast, novices did not show any LSB in any conditions. They also did not show a font-sensitive LSB effect in perceiving Chinese characters, suggesting that the font-sensitive LSB effect we observed among Chinese readers was not simply due to the differences in feature between Ming and Feng fonts which possibly cause different levels of asymmetry; instead, it was related to the perceivers’ familiarity with the fonts.

Nevertheless, in perceiving shared characters, only simplified Chinese readers demonstrated a font-sensitive LSB effect but not traditional Chinese readers, although they were both proficient readers of shared characters as revealed by their reading performance. One plausible explanation is that the font-sensitive LSB effect may also depend on the visual complexity of the characters that the readers are typically exposed to. In our stimuli, the traditional characters were more complex than the simplified and shared characters, while the latter two were matched in terms of visual complexity. According to a Chinese character database analysis [29], the average number of strokes of the most commonly used 3,500 Chinese characters in the simplified script was 9.78 (*SE* = .06), significantly lower than the average of 11.74 (*SE* = .07) in the traditional script (*F*(1, 6998) = 419.38, *p <* .01, *η*_*p*_^*2*^ = .12). Also, when we examined the number of strokes of shared, simplified, and traditional characters in the database separately, traditional characters (*M* = 14.86, *SE* = .07) are more complex than simplified (*M* = 9.04, *SE* = .05; *F*(1, 2464) = 1698.38, *p <* .01, *η*_*p*_^*2*^ = .72) and shared (*M* = 10.18, *SE* = .06; *F*(1, 3498) = 1217.88, *p* < .01, *η*_*p*_^*2*^ = .57) characters, whereas simplified and shared characters do not differ significantly (*F*(1, 3498) = 1.41, *p* = .71). Thus, the visual complexity of the shared and simplified characters is similar to each other, and the shared characters used in our stimuli were similar to what simplified Chinese readers were typically exposed to. Consequently, the simplified Chinese readers exhibited the font-sensitive LSB effect for both simplified and shared Chinese characters, whereas the traditional Chinese readers only showed the font-sensitive LSB effect in traditional characters. These effects suggest that the font-sensitive LSB effect may depend on both the readers’ expertise of the characters and the visual complexity of the characters the readers are typically exposed to.

One important issue in perceptual expertise research is whether and how one’s expertise in one domain can be generalized to another domain. The success of transfer of LSB but not the font-sensitive LSB effect in the processing of different types of Chinese characters suggest that these two perceptual expertise markers might be tapping expertise at different levels. For the transfer of LSB to take place, the similarities in overall structure between two categories might be essential. In face recognition, LSB is argued to be an indicator of the RH dominance in face processing. Similarly, LSB in Chinese character perception is consistent with the literature of the RH/left visual field advantage in Chinese orthographic processing [30] and the more right-lateralized activation in the visual system in fMRI studies [12]. It has been shown that the RH is better than the left hemisphere in processing low spatial frequency information [31], which is more efficient in revealing overall structure of the perceived stimuli but not the details [32]. Thus, the transfer of LSB might rely more on the similarities in configuration between visual categories.

In contrast, effects of within-category variations such as the font-sensitive LSB effect might involve more processing of local featural information, since exemplars of a category (e.g., exemplars of a characters in different fonts) typically have a similar overall structure but differ in local features. Our results showed that the font-sensitive LSB effect was observed only in characters with the visual complexity the readers are typically exposed to in the script they are mostly familiar with, suggesting that it might be a more specific expertise marker of printed scripts. Sanocki observed that word identification was impaired by mixed font as compared with unified font conditions, and suggested that for expert readers, the surface information of a word such as font might be encoded together with the identity of the letter [33, 34]. Gauthier et al. showed that changes in font regularity of Chinese characters affected Chinese readers’ identification but not English readers’ [18]. These results suggest that expert readers’ word recognition is best tuned with one particular font, resulting in an ‘own font effect’, similar to the phenomenon of ‘own race bias’ (ORB) in face perception: own race faces are better recognized than faces of a less familiar race [35]. The ORB effect is suggested to be due to perceptual narrowing, which may also occur in printed word processing, and the font-sensitive LSB effect may be the result of such mechanism. In addition, Navon argued that the global form of a visual stimulus is unavoidably recognized before the local forms, suggesting that in visual expertise development, acquisition of global structures may typically precede that of local features [22]. Given that simplified and traditional Chinese characters generally share similar global structures but differ in features of local components, effects related to global structural processing in one script may be more readily transferred to the other than those related to local information processing. This may be related to why LSB can be generalized across the two scripts but not the font-sensitive LSB effect.

Note that in the current study, the traditional Chinese readers from Taiwan were marginally older and had more education by one year than the simplified Chinese readers and novices of Chinese as a result of random sampling. This marginal age difference, although small, could be a confounding factor for the current results. More specifically, although similar transfer effects were observed in simplified and traditional Chinese readers, there may be difference between the two groups obscured by this potential confounding factor of age. Future work will examine this possibility.

In conclusion, here we showed that both simplified and traditional Chinese readers demonstrated LSB in perceiving Chinese characters while novices did not, validating that LSB is a perceptual expertise marker for visual object recognition; and more importantly, simplified and traditional Chinese readers did not differ in LSB in the perception of simplified and traditional characters, suggesting that LSB in expert Chinese character processing can be transferred across different scripts with similar overall character structures. Nevertheless, the font-sensitive LSB effect could only be observed in characters with the visual complexity they were exposed to the most often in the script they were familiar with. These effects suggest that LSB, as a perceptual expertise marker, can be transferred to the processing of a less familiar stimulus category with similar global structures. In contrast, effects of within-category variations such as the font-sensitive LSB effect require sensitivity to local feature/exemplar variations that emerges only in highly familiar stimuli. The contrast between these two effects thus suggests that transfer of perceptual expertise effects depends on the level of processing (e.g., global configuration vs. local feature) the effect is involved in.

## Supporting information

S1 Appendix Appendix I_tif.tif. Materials for the left-side bias task.
(TIF)

S1 Dataset Supporting Documents_datasets.zip. (ZIP)
