# Supplementary figures and images for: Transfer of the left-side bias effect in perceptual expertise: The case of simplified and traditional Chinese character recognition
Source: PLoS One. 2018 Apr 2;13(4):e0194405. doi: 10.1371/journal.pone.0194405 (PMC5880342; doi:10.1371/journal.pone.0194405)

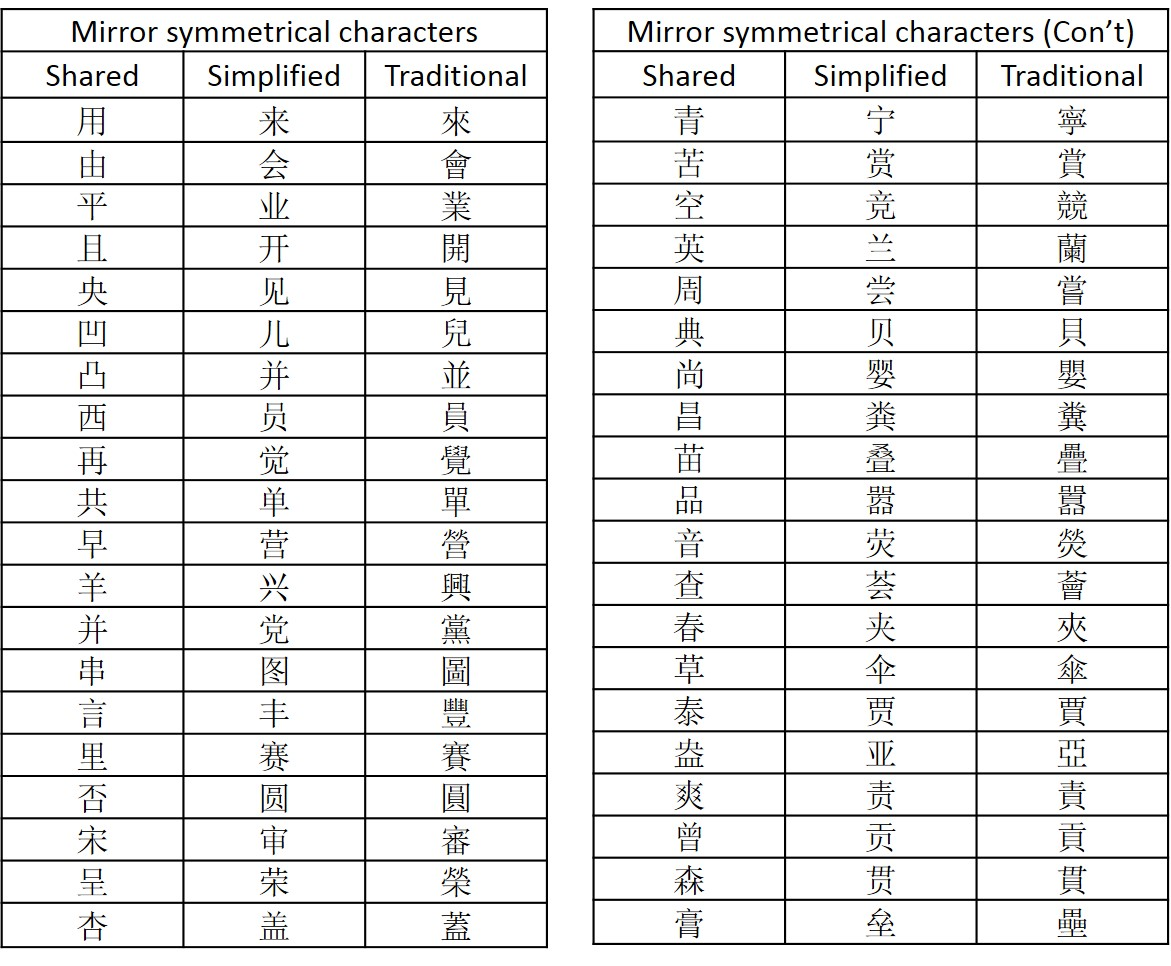

Supplement: S1 Appendix — Materials for the left-side bias task. (TIF) [file pone.0194405.s001.tif]
