# Supplementary material for: Transfer of the left-side bias effect in perceptual expertise: The case of simplified and traditional Chinese character recognition
Source: PLoS One. 2018 Apr 2;13(4):e0194405. doi: 10.1371/journal.pone.0194405 (PMC5880342; doi:10.1371/journal.pone.0194405)
Supplement: S1 Dataset — (ZIP) [file Ethics approval_EA220114(A2).pdf › Supporting Documents_PLOSONE/Ethics approval_EA220114(A2).pdf]

THE UNIVERSITY OF HONG KONG

香 港 大 學

May 23, 2017

Dr. Janet Hui-wen Hsiao
Department of Psychology

Dear Dr. Hsiao,

Application for Amendment of an Approved Project
HREC's Reference Number: **EA220114**

Thank you for your application for amendment to the project "How does writing/drawing experience enhance visual expertise?", which was granted ethics approval by the Human Research Ethics Committee (HREC) in January 2014.

2. I am pleased to inform you that the HREC has approved the change of project period. The expiration date of the ethics approval is extended to March 31, 2018.

3. You are reminded that starting from April 1, 2015, the HREC's reference number of your project (i.e. EA220114) has to be shown in all materials sent to potential and actual participants to enable participants to link the materials to an approved project.

Yours sincerely,

Professor J.H. Bacon-Shone
Chairman
Human Research Ethics Committee

c.c. Dr. A.B. Chan, Department of Computer Sciences, City University of Hong Kong
Dr. W.M. Cheung, Faculty of Education

JBS/cw/bw

THE REGISTRY 教 務 處
POKFULAM ROAD, HONG KONG. TEL:(852)2859 2111 FAX:(852)2858 2549

Printed on environmentally friendly paper
